# Bromopyrene Symphony: Synthesis and Characterisation of Isomeric Derivatives at Non-K Region and Nodal Positions for Diverse Functionalisation Strategies

**DOI:** 10.3390/molecules29051131

**Published:** 2024-03-03

**Authors:** Dawid Zych, Martyna Kubis

**Affiliations:** Faculty of Chemistry, University of Opole, Oleska 48, 45-052 Opole, Poland

**Keywords:** bromopyrenes, substitution pattern, bromination reaction, experimental study, photophysical properties

## Abstract

Pyrene, a renowned aromatic hydrocarbon, continues to captivate researchers due to its versatile properties and potential applications across various scientific domains. Among its derivatives, bromopyrenes stand out for their significance in synthetic chemistry, materials science, and environmental studies. The strategic functionalisation of pyrene at non-K region and nodal positions is crucial for expanding its utility, allowing for diverse functionalisation strategies. Bromo-substituted precursors serve as vital intermediates in synthetic routes; however, the substitution pattern of bromoderivatives significantly impacts their subsequent functionalisation and properties, posing challenges in synthesis and purification. Understanding the distinct electronic structure of pyrene is pivotal, dictating the preferential electrophilic aromatic substitution reactions at specific positions. Despite the wealth of literature, contradictions and complexities persist in synthesising suitably substituted bromopyrenes due to the unpredictable nature of substitution reactions. Building upon historical precedents, this study provides a comprehensive overview of bromine introduction in pyrene derivatives, offering optimised synthesis conditions based on laboratory research. Specifically, the synthesis of mono-, di-, tri-, and tetrabromopyrene isomers at non-K positions (1-, 3-, 6-, 8-) and nodal positions (2-, 7-) is systematically explored. By elucidating efficient synthetic methodologies and reaction conditions, this research contributes to advancing the synthesis and functionalisation strategies of pyrene derivatives, unlocking new possibilities for their utilisation in various fields.

## 1. Introduction

Pyrene needs no introduction; its broad and interesting properties, especially its derivatives, have already been demonstrated over the years in many scientific papers [1,2,3,4]. Pyrene and its derivatives can be widely used in various fields, including organic synthesis, materials science, and environmental chemistry. Its unique physical and chemical properties and air stability make it a valuable tool for studying molecular interactions and environmental processes [5]. To obtain pyrene derivatives appropriate for potential applications, it is required to initially functionalize the active sites: non-K position (1-, 3-, 6-, 8-positions), nodal area (2-, 7-positions), and the K-region (4-, 5-, 9-, 10-positions).

**Figure 1:**
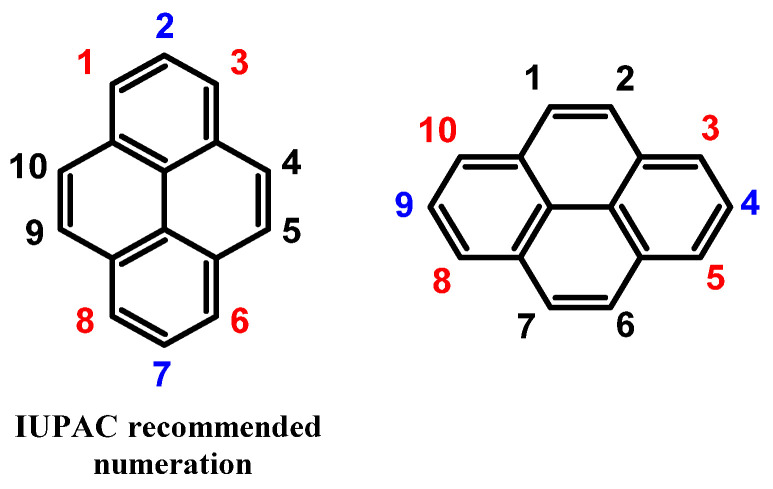
Different locant numerations for pyrene structure (red positions: non-K region; blue positions: nodal area; black positions: K-region).

As time has passed, a concern has emerged regarding the manner in which positions of the pyrene structure are described. The system, which Bally and Scholl first proposed in 1911 [6], served as the foundation for the IUPAC system. The utilisation of this system did not require pyrene to be oriented in a particular way; as a result, diverse numbering was possible (Figure 1) [7]. Patterson enhanced the system that facilitated the designation of names without any ambiguity by considering the numbering of the peripheral atoms and the orientation of the parent polycyclic aromatic hydrocarbon [8]. The Patterson orientation principles, which are essentially equivalent, have been incorporated into the current IUPAC system. Although this system provided improved naming capabilities compared to the foundational method suggested by Bally and Scholl, the chemical community did not readily adopt it. Consequently, other possibilities for numbering the position of pyrene also appeared in the literature, but they were incidental.

Generally, bromosubstituted precursor molecules are essential in synthetic chemistry, because the bromine atom connected to carbon can be transformed into a carbon–carbon or carbon–nitrogen bond based on the protocols of Pd-catalyzed cross-coupling reactions such as Sonogashira [9], Suzuki–Miyaura [10], and the Buchwald–Hartwig amination reaction [11,12]. What is essential is that the substitution pattern of bromoderivatives has a crucial impact on the further functionalisation possibilities and properties of target molecules. Moreover, in addition to the properties and reactivity, the substitution pattern can significantly determine the efficiency and selectivity of the synthetic route and the purification of the desired molecule; the resulting mixture of isomers may be challenging to separate and purify [13].

The unique electronic structure of pyrene predominantly directs electrophilic aromatic substitution reactions towards the 1-, 3-, 6-, and 8-positions, while the 2-, 4-, 5-, 7-, 9-, and 10-positions do not display the same reactivity due to energetic constraints. This discrepancy arises from the fact that the 1-, 3-, 6-, and 8-positions (referred to as the active non-K region) possess approximately 8.8 kcal/mol lower energies compared to the 4-, 5-, 9-, and 10-positions (K-region), whereas the energy difference between the active region and the nodal positions (2- and 7-positions) is notably higher at approximately 20.5 kcal/mol [14]. Consequently, the order of substitution in pyrene can be prioritized as follows: 1 > 8 > 6 > 3 [15]. When reviewing the literature on synthesising suitably substituted bromopyrenes, it becomes apparent that many sources contradict each other, with differences in the reported reaction conditions and resulting products. This can be attributed to the fact that the active positions of pyrene (1-, 3-, 6-, and 8-) display equal reactivity towards substitution, which can occur randomly, resulting in a multitude of products whose composition is influenced by the stoichiometric ratio of the starting materials.

In 1937, Heinrich Vollmann et al. published “*Beiträge zur Kenntnis des Pyrens und seiner Derivate*”, which reported that the bromination of pyrene with a stoichiometric equivalent of bromine in nitrobenzene resulted in 1-bromo-, 1,6-, and 1,8-dibromo isomers, as well as 1,3,6-tribromo- and 1,3,6,8-tetrabromopyrene [16]. Since then, significant advancements have been made in this field, resulting in the development of straightforward methods for obtaining mono-, di-, tri-, and tetrasubstituted pyrenes.

**Figure 2:**
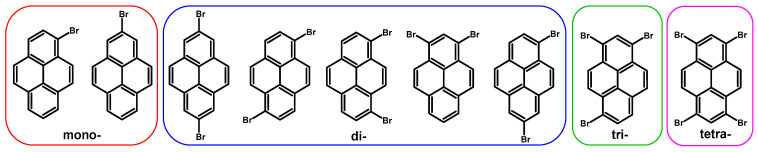
Structure of studied bromoderivatives of pyrene.

While various aspects of pyrene chemistry have been reviewed in the recent literature, this report offers a comprehensive overview of the introduction of bromine with reaction conditions based on our research results where a starting material is pyrene. We present optimal conditions from the laboratory work point of view (time, yields, price, simplicity, purification, and purity of the isomer) for the synthesis of mono-, di-, tri-, and tetrabromo isomer syntheses, including those at non-K positions (1-, 3-, 6-, 8-) and the nodal plane (2-, 7-). Specifically, we report the syntheses of 1-bromopyrene, 2-bromopyrene, 2,7-dibromopyrene, 1,6-dibromopyrene, 1,8-dibromopyrene, 1,3-dibromopyrene, 1,7-dibromopyrene, 1,3,6-tribromopyrene, and 1,3,6,8-tetrabromopyrene (Figure 2).

## 2. Introduction of Bromine

Working with pyrene requires the synthesis of appropriate bromoderivatives. Given the extensive literature on pyrene, selecting the most suitable method to obtain the desired isomer while considering factors such as time, yield, cost, simplicity, purification, and purity can be challenging. Bromination of pyrene can occur at various positions on the pyrene ring, including the non-K regions (1-, 3-, 6-, 8-positions) and the nodal plane (2-, 7-positions). In non-K regions, bromination reactions are typically conducted using a bromine source such as molecular bromine (Br_2_), a brominating agent like *N*-bromosuccinimide (NBS), or a bromine–hydrogen peroxide system (Br_2_/H_2_O_2_). The conditions for bromination may vary depending on the specific isomer being synthesised and can involve solvents, catalysts, or other additives. Conversely, introducing bromine at nodal positions requires initial Ir-catalyzed borylation, which is sterically driven, followed by halogenation. In the following paragraph, we present literature-reported conditions along with yields of products, as described by various authors. Furthermore, drawing from our laboratory experience, we detail the synthesis procedure for bromopyrenes, including necessary modifications (described as Procedure for the synthesis of...). Additionally, NMR spectra were obtained for all products except for 1,3,6,8-tetrabromopyrene, facilitating easy comparison of the particular isomers.

### 2.1. 1,3,6,8-Tetrabromopyrene

**Figure 3:**
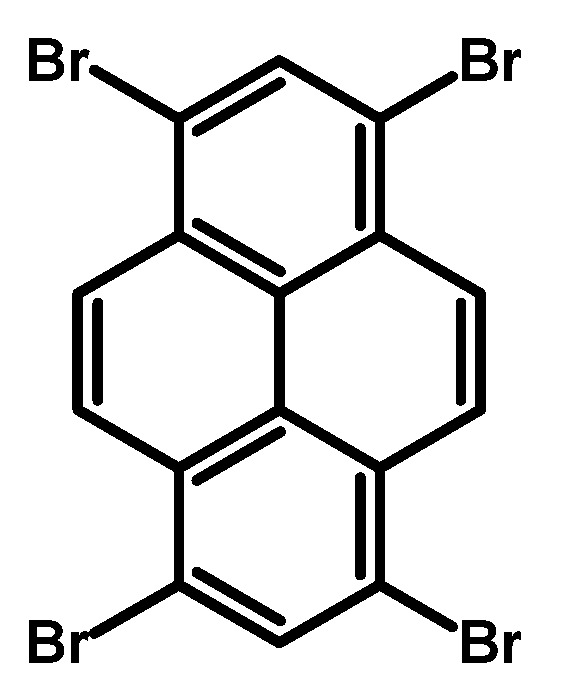
1,3,6,8-Tetrabromopyrene.

In 1937, Vollmann first reported the synthesis of 1,3,6,8-tetrabromopyrene (Figure 3), originally designated as 3,5,8,10-tetrabromopyrene. The synthesis involved brominating pyrene with bromine in nitrobenzene (PhNO_2_), followed by heating at 120 °C for 2 h and an additional 2 h at 120–130 °C [16]. After cooling to 50 °C, the solid was filtered and washed with ethanol, resulting in a 94–96% product yield as yellowish needles.

Since Vollmann’s work, 1,3,6,8-tetrabromopyrene has found applications in numerous reactions, and reported synthesis procedures are based on bromination with bromine in a nitrobenzene solution of pyrene (Table 1). Generally, reactions at 120 °C with shorter durations (2–4 h) consistently yield high percentages (94–99%), suggesting a relatively fast and efficient bromination process under these conditions. Longer reaction times at 120 °C (12–16 h) also result in high yields (96–98%), indicating that the reaction continues to completion with extended time. Higher temperatures (160 °C) show a slight decrease in yield (90%), while the 80 °C reaction for 12 h yields a commendable 92%. The use of nitrobenzene as a solvent remains consistent, emphasising its reliability.

**Procedure for the Synthesis of 1,3,6,8-Tetrabromopyrene:** Pyrene (10.00 g, 49.44 mmol) and nitrobenzene (200 mL) were combined in a three-necked round-bottom flask, to which bromine (34.77 g, 11.14 mL, 217.55 mmol) was added dropwise. The resulting mixture was heated at 120 °C overnight under a nitrogen atmosphere. Subsequently, it was allowed to cool to room temperature, followed by filtration and washing with ethanol and diethyl ether. The product obtained was as light green solid (25.09 g, 98% yield).

### 2.2. 1-Bromopyrene

**Figure 4:**
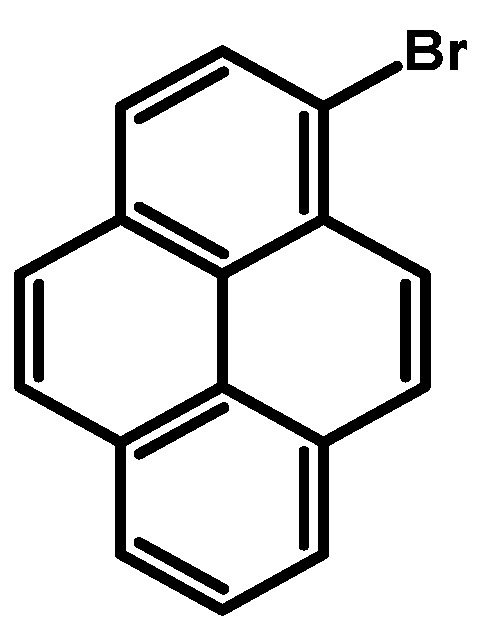
1-Bromopyrene.

In 1937, Lock reported the synthesis of 1-bromopyrene (Figure 4) (originally described as 3-bromopyrene) by the bromination of pyrene [23]. The synthesis involved brominating a pyrene solution in carbon tetrachloride (CCl_4_) using a bromine solution in carbon tetrachloride for 2 h and stirring until the red solution turned yellow again, followed by extraction with water. The obtained solid was dissolved in ethanol, and after cooling, the product was obtained with a 71% yield as yellow crystals.

Subsequent studies have employed analogous methodologies, utilising reagents such as NBS or NBS with an addition, HBr with H_2_O_2_, and benzyltrimethylammonium tribromide (BTMABr_3_) (Table 2). Some of the proposed methods necessitate the use of column chromatography, which imposes a limitation on the reaction scale. A comprehensive analysis of bromination reactions reveals significant correlations between reaction conditions and yields. Notably, higher concentrations of HBr (48%) tend to result in slightly diminished yields, suggesting the presence of an optimal concentration range for this brominating agent. Longer reaction times at room temperature generally correlate with lower yields, though there are exceptions, such as the 24-hour reaction with HBr 48% and H_2_O_2_ 30%, that challenge this trend. Dichloromethane (CH_2_Cl_2_) consistently emerges as a common solvent, yielding favorable results across various brominating agents. Dimethylformamide (DMF) also consistently demonstrates high yields. The influence of reaction temperature is evident, with reactions conducted at lower temperatures (−40 °C) yielding promising results. *N*-bromosuccinimide (NBS), both alone and with additives, consistently produces high yields, as does HBr in conjunction with H_2_O_2_ and Br_2_ under specific conditions. Darkness during the reaction shows varying effects on yields, which are particularly evident in NBS reactions. Additionally, the incorporation of additives such as benzoyl peroxide and unique reagents like 1-phenyl-3,3-dimethyl-1,3-dihydrobenzo[*c*][*1*,*2*]oxaselenol-1-ium tetrafluoroborate positively influences yields. Furthermore, the utilisation of tetrabutylammonium bromide (BTMABr_3_) in combination with CaCO_3_ in a dichloromethane/methanol mixture results in a moderate yield, while its application with ZnCl_2_ in acetic acid leads to a comparatively lower yield. Among all reported procedures, the method presented by M. Schulze and co-workers stands out due to its successful application on a scale of 20 g, using low-cost, commercially available pyrene of technical quality as the starting material [24].

**Procedure for the Synthesis of 1-Bromopyrene:** Pyrene (10.00 g, 49.44 mmol) and a mixture of MeOH/Et_2_O (125 mL, 1:1 *v/v*) were combined in a three-necked round-bottom flask, to which HBr (48% *w/w* aq solution, 9.17 g, 6.15 mL, 54.39 mmol) was added dropwise. The resulting mixture was cooled to 15 °C using an ice-water bath and stirred for 10 min, followed by dropwise addition of H_2_O_2_ (30% *w/w* aq solution, 5.89 g, 5.30 mL, 51.92 mmol) over 30 min. The mixture was stirred overnight under a nitrogen atmosphere. Subsequently, the precipitate was filtrated and washed with a small amount of cold ethanol and diethyl ether. Dichloromethane (100 mL) was added to the filtrate and extracted twice with water. The solvent was evaporated using a rotary evaporator, and the residue was dissolved in hot hexane and placed in a refrigerator overnight. Precipitate was collected by filtration and mixed with the previously obtained solid. The process of dissolving in a small amount of hot hexane was repeated, and the mixture was placed in a refrigerator overnight. After filtration, the product was obtained as a pale yellow solid (10.15 g, 73% yield). ^1^H NMR (400 MHz, CDCl_3_) δ 8.42 (d, *J =* 9.2 Hz, 1H), 8.24–8.18 (m, 3H), 8.15 (d, *J =* 9.2 Hz, 1H), 8.09–8.02 (m, 2H), 8.02–7.96 (m, 2H).

### 2.3. 1,6-Dibromopyrene and 1,8-Dibromopyrene

**Figure 5:**
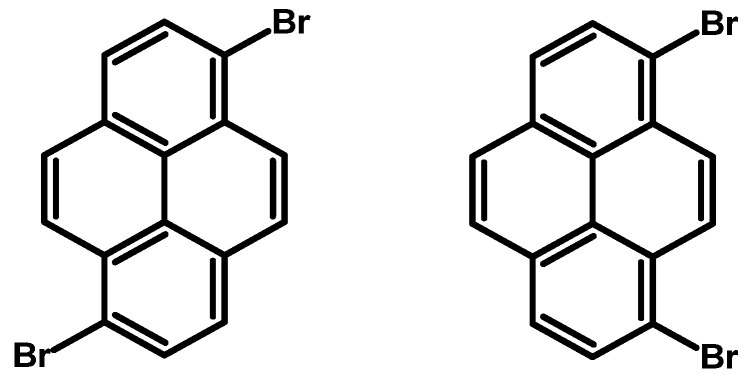
1,6-Dibromopyrene and 1,8-dibromopyrene.

The desire to synthesise 1,6- and 1,8-dibromopyrene (Figure 5) dates back to the 1970s, when J. Grimshaw and J. Trocha-Grimshaw developed a method for their synthesis and separation. This procedure involved gradually adding a bromine solution in carbon tetrachloride (CCl_4_) to a pyrene solution in the same solvent. After stirring overnight, the resultant isomers were isolated and separated by crystallization using either toluene or a combination of benzene and hexane. This yielded the 1,6-isomer and 1,8-isomer as beige solids, with a 44% and 45% yield, respectively [55].

Throughout the years, researchers have explored various solvents, brominating agents, and reaction conditions. Most documented approaches have focused on obtaining the 1,6-isomer, as outlined in Table 3. Notably, the yield of reaction with Br_2_ varies depending on reaction time and conditions, while using 1,3-dibromo-5,5-dimethylhydantoin (DBMH) in CH_2_Cl_2_ for 1 h achieves a high yield of a mixture of isomers—97%. Solvent choices, such as CH_2_Cl_2_ and CHCl_3_, affect yields, with CCl_4_ at 110 °C consistently producing higher yields. Temperature plays a crucial role, as reactions at room temperature yield diverse outcomes, while higher temperatures, such as 110 °C, lead to increased yields in some cases. Longer reaction times generally enhance yields but exceptions exist, such as DBMH in CH_2_Cl_2_ for 1 h. Specific reagent combinations, like benzyltrimethylammonium tribromide (BTMABr_3_) + ZnCl_2_ in CH_2_Cl_2_/MeOH for 16 h, result in quantitative yields. The solvent CS_2_, in the presence of Br_2_, yields 15% with a notable co-product yield of 85%. Darkness conditions in CCl_4_ at 110 °C for 12 h may influence a yield of 63%.

Another method described in the literature for synthesising 1,6- and 1,8-dibromopyrene involves using 1-bromopyrene as the starting material (Table 4). Two papers in the literature detail the reaction conditions. In the initial experiment, a combination of KBr/NaClO in HCl and MeOH solution was employed, resulting in a mixture of products with a yield of 43%. Conversely, in the second scenario, the use of bromine in dichloromethane resulted in the successful synthesis of pure dibromopyrenes, with each isomer yielding approximately 35%.

**Procedure for the Synthesis of 1,6-Dibromopyrene and 1,8-Dibromopyrene:** Pyrene (10.00 g, 49.44 mmol) was combined with carbon tetrachloride (250 mL) in a three-necked round-bottom flask. Bromine (15.80 g, 5.07 mL, 98.89 mmol) was added dropwise over five h under a nitrogen atmosphere. The resulting mixture was stirred overnight. The precipitate formed was then filtered and washed with diethyl ether and hexane. The obtained solid underwent fractional crystallization from toluene, resulting in the initial formation of the less soluble 1,6-dibromopyrene, which crystallized in needle-like structures. The crystallisation process was repeated using toluene. The products were obtained as beige solids, 1,6-dibromopyrene (7.12 g, 40% yield), and 1,8-dibromopyrene (6.23 g, 35% yield). Importantly, due to cost and environmental considerations, the carbon tetrachloride used after filtration was washed three times with water, dried with magnesium sulphate, and then distilled. This purified solvent was utilised in subsequent bromination reactions. Furthermore, the mixture of 1,6- and 1,8-dibromopyrenes obtained from the last crystallization step was employed to synthesise 1,3,6-tribromopyrene. **1,6-Dibromopyrene**: ^1^H NMR (400 MHz, CDCl_3_) δ 8.46 (d, *J =* 9.2 Hz, 2H), 8.27 (d, *J =* 8.2 Hz, 2H), 8.12 (d, *J =* 9.2 Hz, 2H), 8.06 (d, *J =* 8.2 Hz, 2H). **1,8-Dibromopyrene**: ^1^H NMR (400 MHz, CDCl_3_) δ 8.49 (s, 1H), 8.42 (d, *J =* 9.2 Hz, 1H), 8.25 (d, *J =* 8.1 Hz, 2H), 8.08 (d, *J =* 9.2 Hz, 1H), 8.04–8.01 (m, 1H), 8.01 (d, *J =* 3.1 Hz, 2H).

### 2.4. 1,3-Dibromopyrene

**Figure 6:**
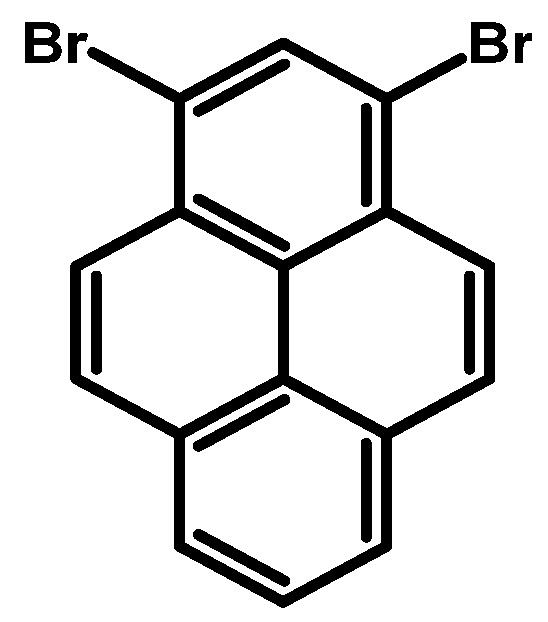
1,3-Dibromopyrene.

Literature on the subject of 1,3-dibromopyrene (Figure 6) is notably scarce due to the challenge of substituting the pyrene structure, which exhibits a preference for electrophilic substitution at the 1,6- and 1,8- positions rather than the 1,3-positions of pyrene. Spectroscopic analysis determined that this isomer is present as a byproduct (with a yield of 3%) of the bromination reaction using bromine in a dichloromethane solution [75].

In 1972, Yu. E. Gerasimenko et al. reported the synthesis of 1,3-dibromopyrene through the decarboxylation reaction of 6,8-dibromo-2-pyrenecarboxylic acid (Table 5) [77]. According to the described protocol, the authors utilised 0.30 g of carboxylic acid, which was dissolved in 20 mL of DMF and 50 mL of H_2_O. The resulting mixture was boiled for 1 h. Subsequently, the obtained dry solid was mixed with 0.20 g of calcium oxide and 0.32 g of calcium hydroxide, and the mixture underwent dry distillation. The target molecule, 1,3-dibromopyrene, was sublimated and obtained as colourless needles, yielding 0.025 g (9.3%).

The alternative approach to obtaining 1,3-dibromopyrene, as presented by Yu. E. Gerasimenko et al., involved the exchange of the carboxylic group with an amine group, followed by the Sandmeyer reaction, resulting in 1,3-dibromopyrene (Table 6) with a yield of 19.6%.

Another synthesis method was described by T. Nielsen et al., wherein 1,3-dibromopyrene was prepared from 1,3-dibromo-7-pyrenecarboxylic acid, previously obtained through the alkaline hydrolysis of methyl 1,3-dibromopyrene-2-carboxylate. The intermediate was subjected to a decarboxylation reaction with copper powder in boiling quinoline [78]. Notably, the authors utilised 230 g of substrate, resulting in 120 mg of 1,3-dibromopyrene.

In the context of a critical analysis of the synthesis possibility of 1,3-dibromopyrene, 2-pyrenecarboxylic acid was synthesised via multistep synthetic routes starting from pyrene [79], followed by bromination in nitrobenzene. Although the decarboxylation reaction was attempted multiple times, and alternative decarboxylation methods were explored by us according to the protocols of described in the literature for the decarboxylation of carboxylic acids of arenes, the desired product was not obtained (Table 7).

Due to the intricate synthesis of 1,3-dibromopyrene, an approach involving the acylation of pyrene allows for obtaining 1,3-disubstituted pyrene, albeit with a *tert*-butyl group at position 7. Incorporation of the *tert*-butyl group can be achieved via a Friedel–Crafts reaction involving *tert*-butyl chloride and AlCl_3_ as a catalyst under various solvent and reaction condition setups. Utilising CH_2_Cl_2_ as the solvent at room temperature for 3 h resulted in an 82–84% yield of 2-*tert*-butylpyrene [83,84]. However, altering the reaction conditions to a temperature range of 0 °C to room temperature within the same time frame led to varying yields, ranging from 65% to 100% [48,85,86,87,88,89,90,91,92]. Remarkably, refluxing the reaction mixture in CS_2_ yielded a high percentage yield at 92% [93].

Regarding the applied amount of brominating agent, 1,3-dibromo-7-*tert*-butylpyrene (Table 8) or 1-bromo-7-*tert*-butylpyrene (Table 9) can be obtained.

For 1-bromo-7-*tert*-butylpyrene, employing Br_2_ in CH_2_Cl_2_ solvent resulted in yields ranging from 72% to 88% across temperatures ranging from −78 °C to room temperature, with longer reaction times generally leading to higher yields. Additionally, the addition of iron to Br_2_ in CH_2_Cl_2_ solvent resulted in 83% yield at temperatures ranging from 0 °C to 28 °C for 5 h. Conversely, utilising NBS in THF solvent provided the highest yield of 94% at temperatures ranging from 0 °C to room temperature overnight.

In the case of 1,3-dibromo-7-*tert*-butylpyrene, using the brominating agent Br_2_ in carbon tetrachloride (CCl_4_) at room temperature for 16 h yielded 68%, while in dichloromethane (CH_2_Cl_2_) at −78 °C, the yield was 89%. The addition of iron alongside Br_2_ in CH_2_Cl_2_ over a temperature range from 0 °C to 28 °C for 5 h resulted in a reduced yield of 35%. In contrast, utilising *N*-bromosuccinimide (NBS) in tetrahydrofuran (THF) at 30 °C overnight provided a notably high yield of 91%. Moreover, employing BTMABr_3_ with calcium carbonate (CaCO_3_) in CH_2_Cl_2_/methanol (MeOH) at 0 °C for 1 h followed by room temperature overnight yielded 76%. Similarly, using BTMABr_3_ in CH_2_Cl_2_ at temperatures ranging from 0 °C to room temperature overnight also yielded 76%.

**Procedure for the Synthesis of 1,3-dibromo-7-*tert*-butylpyrene:** 2-*Tert*-butylpyrene (1.00 g, 3.87 mmol) was combined with dichloromethane (40 mL) in a three-necked round-bottom flask. Bromine (1.24 g, 0.40 mL, 7.74 mmol), dissolved in dichloromethane (40 mL), was added dropwise at −78 °C under a nitrogen atmosphere. The reaction mixture was allowed to slowly warm to room temperature and stirred overnight. Afterwards, the organic layer was washed successively with a sodium thiosulfate (0.3 M) solution and water. The solvent was then removed under reduced pressure using a rotary evaporator. The resulting residue was dissolved in hexane, leading to the crystallisation of the product. The precipitate was collected by filtration, yielding a white–silver solid (1.42 g, 88% yield). ^1^H NMR (400 MHz, CDCl_3_) δ 8.44 (s, 1H), 8.34 (d, *J =* 9.2 Hz, 2H), 8.29 (s, 2H), 8.15 (d, *J =* 9.2 Hz, 2H), 1.60 (s, 9H).

### 2.5. 1,3,6-Tribromopyrene

**Figure 7:**
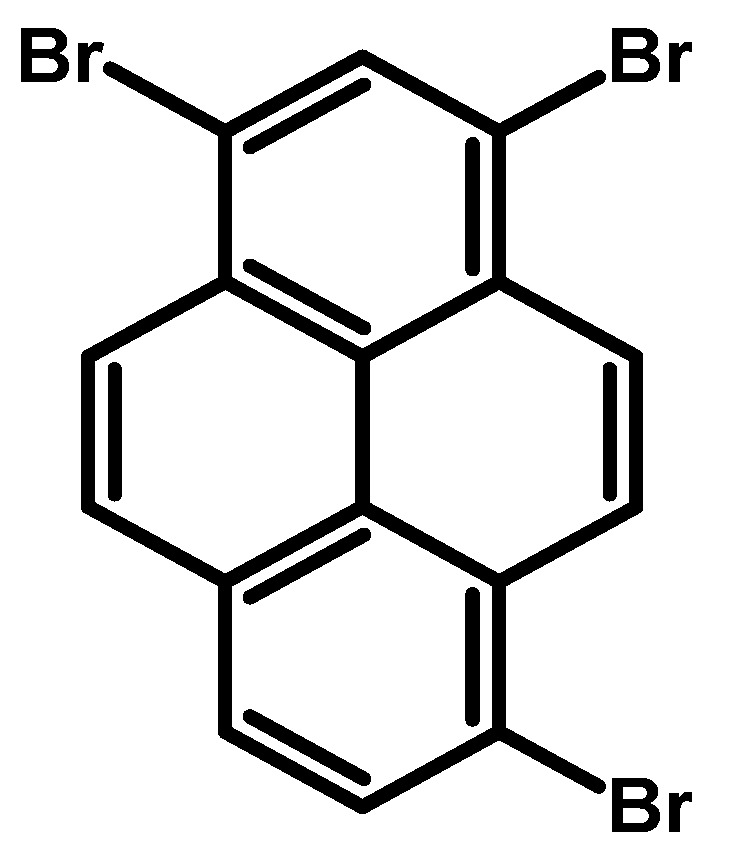
1,3,6-Tribromopyrene.

Following the findings of H. Vollmann et al. in 1937, which detailed the bromination of pyrene with a stoichiometric equivalent of bromine in nitrobenzene, resulting in 1,3,6-tribromopyrene (Figure 7) [16], the synthesis of trisubstituted pyrene was subsequently presented in 1972 by James Grimshaw and J. Trocha-Grimshaw in the same publication, where the authors reported the synthesis of dibromopyrenes (1,6- and 1,8-) and 1-bromopyrene [55]. This method involved dissolving pyrene in carbon tetrachloride, followed by the addition of bromine solution in carbon tetrachloride and stirring the mixture for four days. The resulting precipitate was collected and then extracted with boiling carbon tetrachloride. The residue was subjected to multiple recrystallisations from toluene. However, the final product, obtained with a 14% yield, was found to be contaminated with traces of 1,6-, 1,8-dibromo-, and 1,3,6,8-tetrabromopyrenes.

A similar approach was applied in the following years (Table 10). Conducting the reaction with Br_2_ in nitrobenzene at 80 °C for 12 h led to a significantly higher yield of 87%. However, utilising Br_2_ in nitrotoluene under unspecified conditions resulted in 1,3,6-tribromopyrene, although the authors did not specify the reaction yield.

Another approach to the synthesis described in the literature involves the preparation of 1,3,6-tribromopyrene from pyrene through sequential bromination reactions. Initially, pyrene is reacted with aqueous HBr and H_2_O_2_ to yield a mixture of 1,6- and 1,8-dibromopyrenes via electrophilic aromatic substitution. Subsequently, the mixture of dibromopyrenes was treated with elemental bromine in nitrobenzene, leading to the bromination of the pyrene ring and the formation of 1,3,6-tribromopyrene (Table 11). The reported yield of 82% demonstrates the efficiency of the reaction conditions in converting the dibromopyrene mixture to the desired product.

**Procedure for the Synthesis of 1,3,6-Tribromopyrene:** A mixture of 1,6- and 1,8-dibromopyrene (10.00 g, 27.77 mmol) was combined with nitrobenzene (250 mL) in a three-necked round-bottom flask. Bromine (4.44 g, 1.42 mL, 27.77 mmol) was added dropwise under a nitrogen atmosphere. The resulting mixture was stirred overnight. The precipitate formed was then filtered and washed with diethyl ether and hexane. The obtained solid underwent crystallisation from toluene, resulting in 1,3,6-tribromopyrene as a white solid (9.87 g, 81%). ^1^H NMR (400 MHz, CDCl_3_) δ 8.53 (s, 1H), 8.46 (d, *J =* 9.1 Hz, 2H), 8.27 (d, *J =* 2.4 Hz, 1H), 8.25 (d, *J =* 2.4 Hz, 2H), 8.12 (d, *J =* 9.2 Hz, 2H), 8.06 (d, *J =* 4.8 Hz, 2H), 8.04 (t, *J =* 2.4 Hz, 2H).

### 2.6. 1,7-Dibromopyrene

In 2022, Y. Ahn et al. reported the synthesis of 1,7-dibromopyrene (Table 12). The synthesis involved the substitution of 1-bromopyrene by a borate group at position 7, followed by exchange with bromine. The synthetic route commenced with the dissolution of 2-(6-bromopyren-2-yl)-4,4,5,5-tetramethyl-1,3,2-dioxaborolane, 4,4′-di-*tert*-butyl-2,2′-dipyridyl (dtbpy), and bis(pinacolato)diboron in anhydrous cyclohexane under nitrogen. The reaction mixture, containing 1-bromopyrene and an additional portion of bis(pinacolato)diboron in cyclohexane, was then added and stirred at 70 °C overnight. The resulting crude mixture underwent extraction, drying, and purification by silica gel column chromatography. A subsequent reaction with copper(II) bromide in a water–isopropanol–dimethylformamide solution at a higher temperature (110 °C) resulted in a crude product. The final product was obtained through filtration, washing, and recrystallisation, resulting in a white solid with a 66% yield.

**Procedure for the Synthesis of 1,7-Dibromopyrene:** A solution of [Ir(*μ*-OMe)(cod)]₂ (0.073 g, 0.15 mmol), 4,4′-di-*tert*-butyl-2,2′-dipyridyl (dtbpy) (0.097 g, 0.36 mmol), and bis(pinacolato)diboron (0.198 g, 0.78 mmol) in hexane (10 mL) was prepared in a Schlenk flask under a nitrogen atmosphere. The mixture was stirred for 10 min. Then, a solution of 1-bromopyrene (5.00 g, 17.78 mmol) and bis(pinacolato)diboron (4.77 g, 18.78 mmol) in hexane (20 mL) was added to the reaction mixture. The Schlenk flask was purged with nitrogen, and the resulting mixture was stirred at 70 °C overnight. The crude product was subsequently extracted with chloroform and water. The organic layer was separated, and the solvent was removed under reduced pressure using a rotary evaporator. The resulting residue was dissolved in dichloromethane and passed through a layer of silica gel. The solvent was then removed under reduced pressure, and the obtained yellow residue was dissolved in a mixture of methanol and tetrahydrofuran (MeOH/THF, 60 mL, 3:1 *v/v*). Then, a solution of copper(II) bromide (19.86 g, 88.9 mmol) in 30 mL of water was added to the solution. The mixture was stirred overnight at 90 °C under a nitrogen atmosphere. The resulting product was filtered and washed successively with water, diethyl ether, and hexane. The obtained precipitate was then purified by silica gel column chromatography using chloroform as the eluent to obtain a solid, which was crystallised from acetonitrile to yield the desired compound as a white solid (1.98 g, 31%). ^1^H NMR (400 MHz, CDCl_3_) δ 8.53 (s, 1H), 8.45 (d, *J =* 9.2 Hz, 1H), 8.21 (d, *J =* 8.0 Hz, 1H), 8.13 (d, *J =* 2.5 Hz, 1H), 8.05 (s, 1H), 7.89 (d, *J =* 9.2 Hz, 1H), 7.69 (d, *J =* 9.2 Hz, 1H), 7.63 (d, *J =* 9.3 Hz, 1H).

### 2.7. 2-Bromopyrene and 2,7-Dibromopyrene

**Figure 8:**
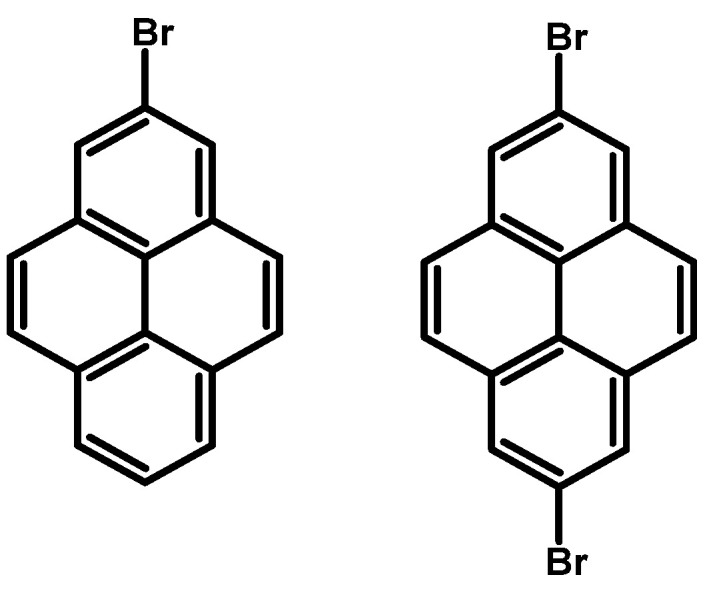
2-Bromo and 2,7-dibromopyrene.

In 1965, A. Streitwieser and co-workers reported for the first time in the literature 2-bromopyrene, whereas 2,7-dibromopyrene was presented 21 years later in 1986 by H. Lee and R. G. Harvey (Figure 8).

The synthesis of 2-bromopyrene was conducted through a multi-step process, initiated by the reaction of sodium nitrite, sulfuric acid, and water, followed by the addition of urea and subsequent treatment with mercury(II) bromide and potassium bromide (Table 13). This sequence of reactions was carried out over a total duration of 4 h, yielding salt of 2-bromopyrene (C_16_H_9_BrN_2_*HgBr_2_) with an efficiency of 120%. The obtained intermediate was employed in a reaction with potassium bromide at higher temperature conditions (120 °C) for 0.5 h, yielding 2-bromopyrene with an efficiency of 32%.

The synthesis of 2,7-dibromopyrene was accomplished in two steps with high efficiency (Table 14). Initially, bromination of 4,5,9,10-tetrahydropyrene was conducted at room temperature overnight using bromine in the presence of iron(III) chloride hydrate as a catalyst and water as a solvent. This step resulted in a remarkable yield of 99%, yielding 2,7-dibromo-4,5,9,10-tetrahydropyrene. Subsequently, the obtained intermediate was subjected to further bromination under room temperature conditions for 4 h, employing bromine in the presence of carbon disulfide, yielding 2,7-dibromopyrene with a conversion of 73%.

In the following years, 2-bromopyrene and 2,7-dibromopyrene were synthesised starting from 4,5,9,10-tetrahydropyrene, followed by mono- [103,104] or dibromination [105,106] and aromatisation, resulting in products with yields up to 93%. However, a major limitation of this synthesis approach is connected with the substrate—4,5,9,10-tetrahydropyrene. Its synthesis, starting from pyrene, requires a high-pressure autoclave, hydrogen, and palladium catalyst, making the commercially available 4,5,9,10-tetrahydropyrene quite expensive (about 280 USD/1 g). Consequently, alternative methods for the synthesis of 2-bromo and 2,7-dibromopyrene were explored [107].

Ir-catalyzed borylation has seen significant advancements over the past twenty years and has been successfully utilised in the production of pyrene derivatives, typically occurring at locations that have lower steric hindrance and higher proton acidity, in contrast to the conventional electrophilic substitution. Borylation with 2.15 Eq. (in the case of disubstituted) or 1.15 Eq. (in the case of monosubstituted) of bis(pinacolato)diboron (B_2_pin_2_) at positions 2 and 2,7- was conducted in the presence of catalyst bis(1,5-cyclooctadiene)diiridium(I) dichloride ([Ir(*μ*-OMe)cod)]_2_) and 4,4’-di-*tert*-butyl-2,2′-bipyridine (dtbpy) in solvents such as THF or hexane (Table 15). Reaction temperatures were maintained at 80 °C for a duration of 16 h. Yield percentages were determined, with the disubstituted pyrene reaction yielding between 81% and 94% and the monosubstituted product yielding 65%.

Subsequently, we repeated the protocol of direct Ir-catalyzed borylation at positions 2 and 7. Applying 1.15 Eq. B_2_pin_2_ resulted in disubstituted pyrene at positions 2 and 7 with 4,4,5,5-tetramethyl-1,3,2-dioxaborolane groups instead of the monosubstituted pyrene reported in the literature. However, modifying the literature procedure by decreasing the amount of applied B_2_pin_2_ to 0.6 Eq. resulted in the formation of 2-(4,4,5,5-tetramethyl-1,3,2-dioxaborolan-2-yl)pyrene with a yield of 52%.

Boroorganic molecules can be used in the synthesis of bromo analogues. In the case of 2-bromopyrene, reported in the literature, the first method utilises *N*-Bromosuccinimide (NBS) in chloroform (CHCl_3_) at temperatures ranging from 15–20 °C over a 24-h period, resulting in a high yield of 96% (Table 16). In contrast, the second approach involves the use of copper(II) bromide (CuBr_2_) in a mixture of methanol and water (MeOH/H_2_O in a 1:1, *v/v*) at a higher temperature of 90 °C for 16 h, yielding 83%.

In the case of 2,7-disubstituted pyrene by boroorganic groups, reported experiments in the literature were conducted using a solvent mixture of THF and MeOH in different ratios, with the addition of water (Table 17). Reaction temperatures were maintained at 90 °C, with varying reaction times. The first condition employed THF/MeOH (1:3) with the inclusion of water and an overnight reaction time, yielding a moderate 64% yield. Subsequent experiments adjusted the solvent composition to include more water and varied the reaction times. A shift to THF/MeOH/H_2_O (1:3:3) with a 16 h reaction time led to a slight improvement in yield to 70%. Remarkably, reducing the reaction time to 12 h under the same solvent composition resulted in a significant enhancement in yield, achieving an impressive 98% yield.

**Procedure for the Synthesis of 2-(4,4,5,5-tetramethyl-1,3,2-dioxaborolan-2-yl)pyrene and 2,7-bis(4,4,5,5-tetramethyl-1,3,2-dioxaborolan-2-yl)pyrene:** A solution of [Ir(*μ*-OMe)(cod)]₂ (0.044 g, 0.09 mmol), 4,4′-di-*tert*-butyl-2,2′-dipyridyl (dtbpy) (0.048 g, 0.18 mmol), and bis(pinacolato)diboron (0.102 g, 0.4 mmol) in hexane (5 mL) was prepared in a Schlenk flask under a nitrogen atmosphere. The mixture was stirred for 10 min. Subsequently, a solution of pyrene (2.00 g, 9.89 mmol) and bis(pinacolato)diboron (for monosubstituted: 1.40 g, 5.53 mmol or for disubstituted: 2.79 g, 11.00 mmol) in hexane (for monosubstituted: 10 mL or for disubstituted: 20 mL) was added to the reaction mixture. The Schlenk flask was purged with nitrogen, and the resulting mixture was stirred at 70 °C overnight. The crude product was then extracted with chloroform and water. The organic layer was separated, and the solvent was removed under reduced pressure using a rotary evaporator. The resulting residue was purified by column chromatography on silica gel (eluent: hexane:dichloromethane, 1:1, *v/v*). The obtained yellowish oil was mixed with hexane, and the obtained precipitate was filtered. 2-(4,4,5,5-Tetramethyl-1,3,2-dioxaborolan-2-yl)pyrene was obtained as a white solid (1.69 g, 52%) or 2,7-bis(4,4,5,5-tetramethyl-1,3,2-dioxaborolan-2-yl)pyrene was obtained as a white solid (3.77 g, 84%). **2-(4,4,5,5-tetramethyl-1,3,2-dioxaborolan-2-yl)pyrene:** ^1^H NMR (400 MHz, CDCl_3_) δ 8.67 (s, 2H), 8.17 (d, *J =* 7.6 Hz, 2H), 8.12 (d, *J =* 9.0 Hz, 2H), 8.07 (d, *J =* 9.0 Hz, 2H), 8.03 (d, *J =* 7.3 Hz, 1H), 1.48 (s, 12H). ^13^C NMR (101 MHz, CDCl_3_) δ 131.74, 131.45, 130.52, 127.88, 127.39, 126.49, 126.46, 124.95, 124.71, 84.29, 25.13. **2,7-bis(4,4,5,5-tetramethyl-1,3,2-dioxaborolan-2-yl)pyrene:** ^1^H NMR (400 MHz, CDCl_3_) δ 8.62 (s, 4H), 8.09 (s, 4H), 1.46 (s, 24H). ^13^C NMR (101 MHz, CDCl_3_) δ 131.3, 131.0, 127.8, 126.4, 84.3, 25.1.
**Procedure for the Synthesis of 2-Bromopyrene and 2,7-Dibromopyrene:** 2-(4,4,5,5-tetramethyl-1,3,2-dioxaborolan-2-yl)pyrene (0.33 g, 1.00 mmol) or 2,7-bis(4,4,5,5-tetramethyl-1,3,2-dioxaborolan-2-yl)pyrene (0.45 g, 1.00 mmol) was dissolved in a mixture of methanol and tetrahydrofuran (MeOH/THF, 60 mL, 3:1 *v/v*). Then, a solution of copper(II) bromide (for monosubstituted: 2.23 g, 10.00 mmol or for disubstituted: 4.47 g, 20.00 mmol) in 30 mL of water was added to a solution. The mixture was stirred overnight at 90 °C under a nitrogen atmosphere. The resulting product was filtered and washed successively with water, diethyl ether, and hexane. The obtained precipitate was crystallised from hot hexane to yield the desired compound as a beige solid 2-bromopyrene (0.24 g, 86%) and 2,7-dibromopyrene (0.32 g, 89%). **2-Bromopyrene:** ^1^H NMR (400 MHz, CDCl_3_) δ 8.16 (s, 2H), 8.12 (d, *J* = 7.5 Hz, 2H), 8.00–7.94 (m, 2H), 7.83 (d, *J* = 9.0 Hz, 2H), 7.80–7.72 (m, 1H). **2,7-Dibromopyrene:** ^1^H NMR (400 MHz, CDCl_3_) δ 8.31 (s, 4H), 8.01 (s, 4H).

### 2.8. NMR Spectra of Bromopyrenes

In this study, we recorded ^1^H NMR spectra using a Bruker Avance 400 MHz instrument and compared them for a series of brominated compounds dissolved in deuterated chloroform (Table 18).

## 3. Conclusions

In conclusion, this study sheds light on the synthesis and characterisation of bromopyrene derivatives, emphasising the strategic functionalisation at non-K region and nodal positions to enable diverse functionalisation strategies. Through systematic exploration and optimisation of synthesis conditions, including reaction time, yields, cost-effectiveness, simplicity, and purification methods, we have provided valuable insights into synthesising mono-, di-, tri-, and tetrabromopyrene isomers. Our findings contribute to advancing the understanding of pyrene chemistry and offer practical guidance for researchers aiming to utilise pyrene derivatives in various scientific applications. By elucidating efficient synthetic methodologies, this research opens up new avenues for developing functionalised pyrene derivatives with tailored properties, facilitating their utilisation in fields such as organic synthesis, materials science, and environmental chemistry.

## Figures and Tables

**Table 1:** Reported reaction conditions for obtaining 1,3,6,8-tetrabromopyrene.

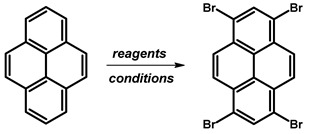				
Brominating Agent	Solvent	Reaction Conditions	Yield [%]	Ref.
Br_2_	PhNO_2_	80 °C, 12 h	92	[17]
		160 °C, 3 h	90	[18]
		120 °C, 16 h	98	[19]
		120 °C, 4 h	94	[20]
		120 °C, 2 h	99	[21]
		120 °C, 12 h	96	[22]

**Table 2:** Reported reaction conditions in obtaining 1-bromopyrene.

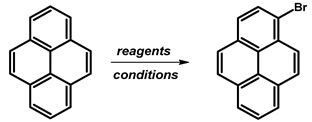				
Brominating Agent	Solvent	Reaction Conditions	Yield [%]	Ref.
HBr 40%, H_2_O_2_ 30%	MeOH/Et_2_O (1:1)	rt, 12 h	96	[25]
HBr 48%, H_2_O_2_ 30%	MeOH/Et_2_O (1:1)	30 °C, 12 h	90	[26]
		rt, 14 h	86	[27]
		rt, 12 h	84.4	[28]
		rt, 12 h	84.7	[29]
		rt, 16 h	77	[24]
		rt, 24 h	90	[30]
HBr 48%, H_2_O_2_ 35%	MeOH/Et_2_O (1:1)	rt, 12 h	95	[31,32]
		rt, 14 h	86	[33]
NBS	DCM	rt, 2 h	95	[34]
		rt, 6 h	90	[35]
		rt, 6 h	88	[36]
NBS, benzoyl peroxide	DMF	rt	96	[37]
NBS	DMF	rt, over a night	94	[38]
		rt, over a night	78	[39]
		rt, 24 h	85	[22,40,41]
		rt	65–70	[42,43]
NBS, *additive 1*	DCM	−40 °C, darkness	82	[44]
NBS, C_28_H_28_Se_2_(BF_4_)_2_	DCM	−40 °C, darkness	95	[45]
NBS, *additive 2*	DCM	−40 °C, 72 h, darkness	91	[46]
NBS, *additive 3*	DCM	rt, 6 h, darkness	85	[47]
Br_2_	DCM	rt, overnight	75	[48]
Br_2_	CCl_4_	rt, overnight	86	[49]
			82	[50]
Br_2_	CHCl_3_	80 °C, 24 h	81	[51]
Br_2_	PhNO_2_	120 °C, reflux, 10 h	-	[52]
BTMABr_3_, CaCO_3_	DCM/MeOH (1:3)	rt, 4 h	80.3	[53]
BTMABr_3_, ZnCl_2_	AcOH	rt, 12 h	67	[54]

*additive 1*—1-[henyl-3,3-dimethyl-1,3-dihydrobenzo[*c*][*1*,*2*]oxaselenol-1-ium tetrafluoroborate; *additive 2*—methyl bis[4-(trifluoromethyl)phenyl]selenonium tetrafluoroborate; *additive 3*—2,2′-di(quinolin-8-yl)-1,1’-spirobi[benzo[*d*][*1*,*2*]selenazole]-3,3′(*2H*,*2′H*)-dione.

**Table 3:** Reported reaction conditions in obtaining 1,6-dibromopyrene and 1,8-dibromopyrene.

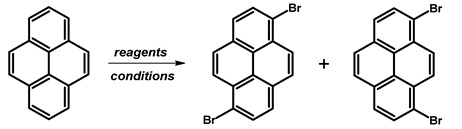					
Brominating Agent	Solvent	Reaction Conditions	Yield [%]		Ref.
			1,6-	1,8-	
Br_2_	CH_2_Cl_2_	rt, 24 h	15	-	[56]
		rt, 2 h	50	-	[57]
		rt, 20 h	25	9	[58]
Br_2_	CHCl_3_	rt, 17 h	33	-	[59,60]
		rt, 24 h	36	-	[61]
		rt, 5 h	14	-	[62]
		rt, 17 h	14	6	[63]
		rt, 5 h	14	6	[63]
		20 °C, 4 h	25	9	[58,64]
		rt, 24 h	32	-	[65]
Br_2_	CCl_4_	110 °C, 12 h, darkness	63	-	[66]
		rt, 16 h	21	-	[67]
		rt, 17 h	44	45	[55]
		rt, 17 h	61	-	[39]
		rt, 24 h	28	13	[68]
		rt, 48 h	38	-	[69]
		rt, 54 h	25	50	[70]
		rt, 12 h	43	-	[71]
Br_2_	CS_2_	rt, 17 h	15	85	[72,73]
DBMH	CH_2_Cl_2_	rt, 1 h	97		[22]
BTMABr_3_ + ZnCl_2_	CH_2_Cl_2_ MeOH	rt, 16 h	quant.		[74]

**Table 4:** Reported reaction conditions in obtaining 1,6- and 1,8-dibromopyrene from 1-bromopyrene.

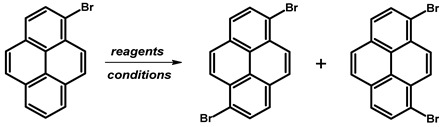					
Brominating Agent	Solvent	Reaction Conditions	Yield [%]		Ref.
			1,6-	1,8-	
KBr + NaClO	HCl, MeOH	rt, 24 h	43		[75]
Br_2_	CH_2_Cl_2_	rt, 6 h	35	36	[76]

**Table 5:** Synthetic route for obtaining 1,3-dibromopyrene.

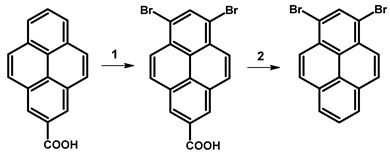					
	Reagents	Solvent	Reaction Conditions	Yield [%]	Ref.
**1**	Br_2_	PhNO_2_	120 °C	80	[77]
**2**	CaO, Ca(OH)_2_	DMF, H2O	reflux	9.3	

**Table 6:** Synthetic route for obtaining 1,3-dibromopyrene through 1,3-dibromo-7-aminopyrene.

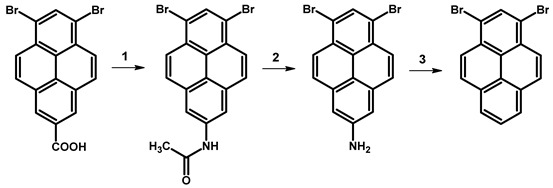				
	Reagents	Reaction Conditions	Yield [%]	Ref.
**1**	PCl_5_, py, NaN_3_, Ac_2_O, H_2_O	100 °C, 1 h	48.5	[77]
**2**	HCl, AcOH	reflux	98	
**3**	NaNO_2_, H_2_SO_4_, AcOH	reflux	19.6	

**Table 7:** Decarboxylation methods for obtaining 1,3-dibromopyrene.

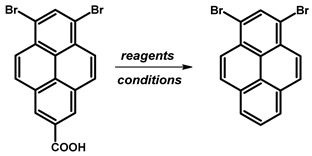			
Substrates	Reaction Conditions	Yield [%]	Ref.
Cu, quinoline	reflux, 1.5 h	≈0	[78]
Cu_2_O, TMEDA, NMP	120 °C, 4 h	≈0	[80]
Cu_2_O, quinoline, NMP	180 °C, 12 h	≈0	[81]
Cu_2_O, 4,7-diphenyl-1,10-phenanthroline, NMP	170 °C, 12 h	≈0	[82]
Cu_2_O, 1,10-phenanthroline, quinoline, NMP	180 °C, 1 h, MW	≈0	[82]

**Table 8:** Reported reaction conditions for obtaining 1-bromo-7-*tert*-butylpyrene.

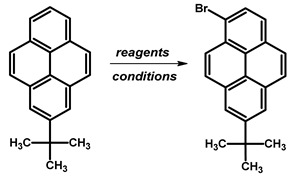				
Brominating Agent	Solvent	Reaction Conditions	Yield [%]	Ref.
Br_2_	CH_2_Cl_2_	−78 °C to rt, overnight	88	[84]
Br_2_	CH_2_Cl_2_	−78 °C to rt, overnight	75	[48,85,89]
Br_2_	CH_2_Cl_2_	−78 °C to rt, 10 h	72	[93]
Br_2_	CH_2_Cl_2_	−78 °C to rt, 14 h	80	[90]
Br_2_, Fe	CH_2_Cl_2_	0 °C to 28 °C, 5 h	83	[86]
BTMABr_3_	CH_2_Cl_2_	0 °C to rt, 3 h	84	[86]
NBS	THF	0 °C to rt, overnight	94	[94]

**Table 9:** Reported reaction conditions for obtaining 1,3-dibromo-7-*tert*-butylpyrene.

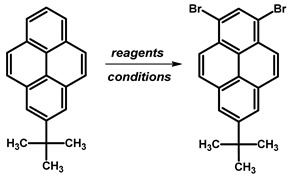				
Brominating Agent	Solvent	Reaction Conditions	Yield [%]	Ref.
Br_2_	CCl_4_	rt, 16 h	68	[83]
Br_2_	CH_2_Cl_2_	−78 °C	89	[89]
Br_2_, Fe	CH_2_Cl_2_	0 °C to 28 °C, 5 h	35	[86]
NBS	THF	30 °C, overnight	91	[65]
BTMABr_3_, CaCO_3_	CH_2_Cl_2_/MeOH	0 °C, 1 h rt, overnight	76	[87,88,91]
BTMABr_3_	CH_2_Cl_2_	0 °C to rt, overnight	76	[86]

**Table 10:** Reported reaction conditions for obtaining 1,3,6-tribromopyrene starting from pyrene.

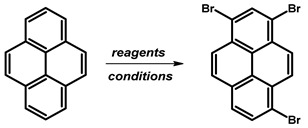				
Brominating Agent	Solvent	Reaction Conditions	Yield [%]	Ref.
Br_2_	PhNO_2_	rt, 96 h	14	[55]
Br_2_	nitrotoluene	-	-	[95,96,97]
Br_2_	PhNO_2_	80 °C, 12 h	87	[98]

**Table 11:** Reported reaction conditions for obtaining 1,3,6-tribromopyrene starting from dibromopyrenes.

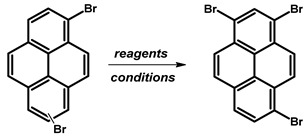				
Brominating Agent	Solvent	Reaction Conditions	Yield [%]	Ref.
Br_2_	PhNO_2_	rt, 12 h	82	[99]

**Table 12:** Reported reaction conditions for obtaining of 1,7-dibromopyrene.

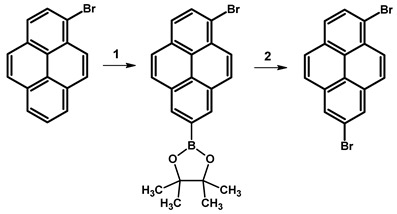					
	Reagents	Solvent	Reaction Conditions	Yield [%]	Ref.
**1**	B_2_pin_2_, [Ir(*μ*-OMe)(cod)]_2_, dtbpy	cyclohexane	70 °C, overnight	22	[100]
**2**	CuBr_2_	isopropanol:DMF (1:1), H_2_O	110 °C, 6 h	66	

**Table 13:** First reported synthesis of 2-bromopyrene.

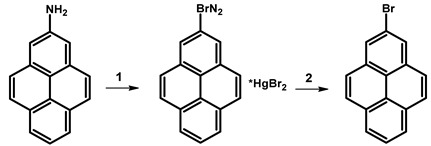				
	Reagents	Reaction Conditions	Yield [%]	Ref.
**1**	NaNO_2_, H_2_SO_4_, H_2_O; CO(NH₂)₂, H_2_O; HgBr_2_, KBr, H_2_O	2 h; 1 h; 1 h	120 (C_16_H_9_BrN_2_*HgBr_2_)	[101]
**2**	KBr	120 °C, 0.5 h	32	

**Table 14:** First reported synthesis of 2,7-dibromopyrene.

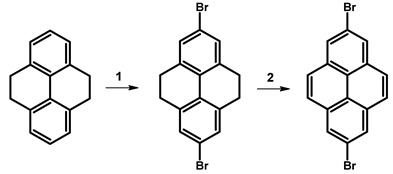				
	Reagents	Reaction Conditions	Yield [%]	Ref.
**1**	Br_2_, FeCl_3_·H_2_O, H_2_O	rt, overnight	99	[102]
**2**	Br_2_, CS_2_	rt, 4 h	73	

**Table 15:** Reported reaction conditions for obtaining boroorganic pyrene derivatives.

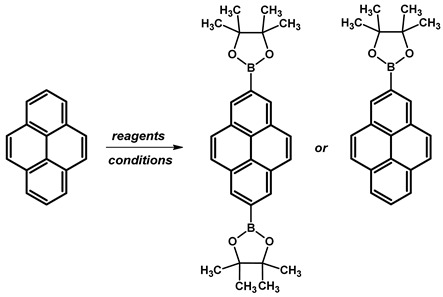				
	Reagents	Reaction Conditions	Yield [%]	Ref.
**2,7-di**	B_2_pin_2_, [Ir(*μ*-OMe)(cod)]_2_, dtbpy, THF	80 °C, 16 h	81	[108]
			94	[109]
**2-mono**	B_2_pin_2_, [Ir(*μ*-OMe)(cod)]_2_, dtbpy, hexane	80 °C, 16 h	65	[109]

**Table 16:** Reported reaction conditions for obtaining 2-bromopyrene.

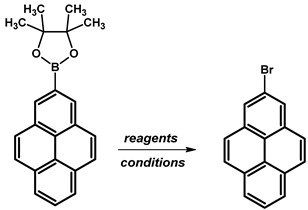				
Brominating Agent	Solvent	Reaction Conditions	Yield [%]	Ref.
NBS	CHCl_3_	15–20 °C, 24 h	96	[110]
CuBr_2_	MeOH/H_2_O (1:1)	90 °C, 16 h	83	[109]

**Table 17:** Reported reaction conditions for obtaining 2,7-dibromopyrene.

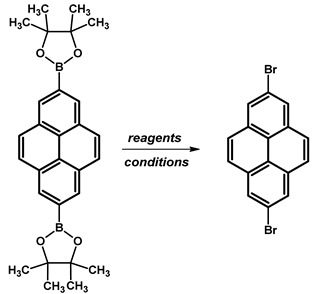				
Brominating Agent	Solvent	Reaction Conditions	Yield [%]	Ref.
CuBr_2_	THF/MeOH (1:3), H_2_O	90 °C, overnight	64	[108]
CuBr_2_	THF/MeOH/H_2_O (1:3:3)	90 °C, 16 h	70	[109]
CuBr_2_	THF/MeOH/H_2_O (1:3:3)	90 °C, 12 h	98	[111]

**Table 18:** NMR spectra comparison of bromopyrenes.

**1,3,6-triBr**	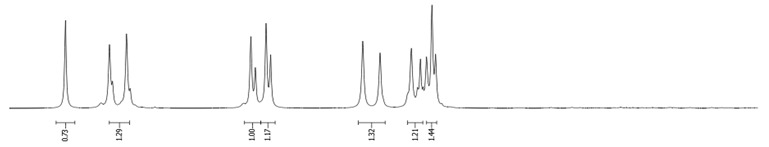
**1,3-diBr-7-*tert*-butyl**	** 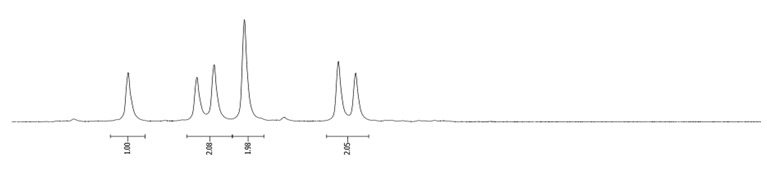 **
**2,7-diBr**	** 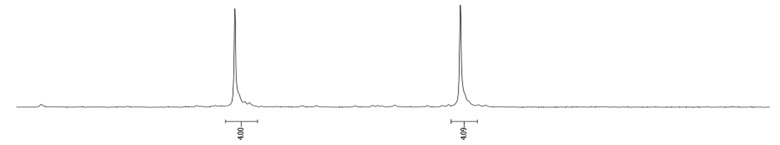 **
**1,7-diBr**	** 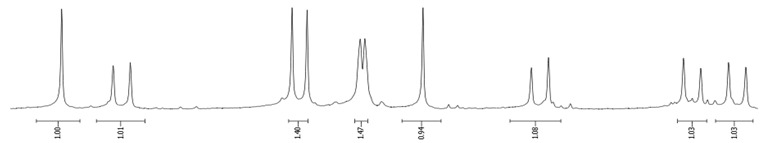 **
**1,8-diBr**	** 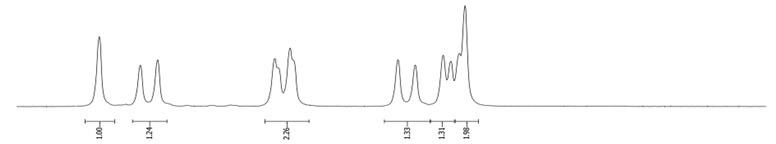 **
**1,6-diBr**	** 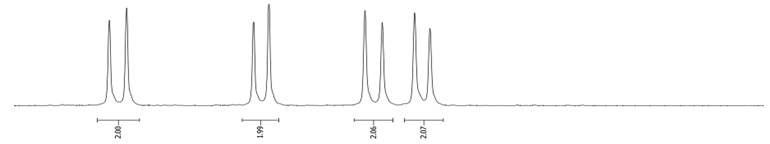 **
**2-Br**	** 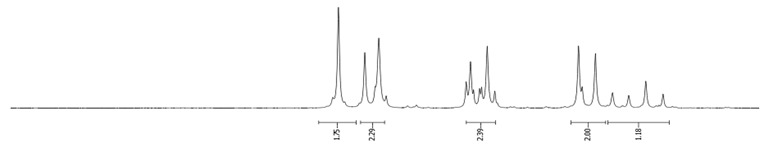 **
**1-Br**	** 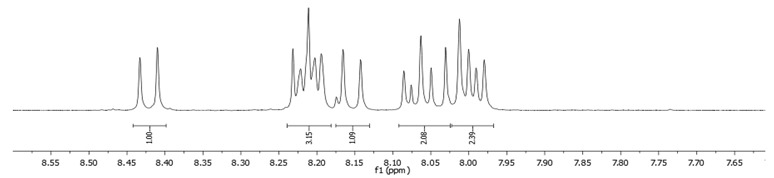 **

## Data Availability

Data are contained within the article.

## References

[1] Kowser Z., Rayhan U., Akther T., Redshaw C., Yamato T. (2021). A Brief Review on Novel Pyrene Based Fluorometric and Colorimetric Chemosensors for the Detection of Cu^2+^. Mater. Chem. Front..

[2] Qutob M., Rafatullah M., Muhammad S.A., Alosaimi A.M., Alorfi H.S., Hussein M.A. (2022). A Review of Pyrene Bioremediation Using Mycobacterium Strains in a Different Matrix. Fermentation.

[3] Figueira-Duarte T.M., Müllen K. (2011). Pyrene-Based Materials for Organic Electronics. Chem. Rev..

[4] Kinik F.P., Ortega-Guerrero A., Ongari D., Ireland C.P., Smit B. (2021). Pyrene-Based Metal Organic Frameworks: From Synthesis to Applications. Chem. Soc. Rev..

[5] Feng X., Hu J.-Y., Redshaw C., Yamato T. (2016). Functionalization of Pyrene To Prepare Luminescent Materials—Typical Examples of Synthetic Methodology. Chem. A Eur. J..

[6] Bally O., Scholl R. (1911). Einwirkung von Glycerin Und Schwefelsäure Auf Amidierte Und Auf Stickstofffreie Verbindungen Der Anthracen-Reihe: Benzanthron Und Seine Reduktionsprodukte, Nebst Bemerkungen Über Namenbildung Und Ortsbezeichnung Hochgegliederter Ringsysteme Der Anthracen-Reihe. Berichte Dtsch. Chem. Ges..

[7] Clar E., Clar E. (1964). Nomenclature of Polycyclic Hydrocarbons. Polycyclic Hydrocarbons: Volume 1.

[8] Patterson A.M. (1925). Proposed international rules for numbering organic ring systems. J. Am. Chem. Soc..

[9] Chinchilla R., Nájera C. (2007). The Sonogashira Reaction:  A Booming Methodology in Synthetic Organic Chemistry. Chem. Rev..

[10] Farhang M., Akbarzadeh A.R., Rabbani M., Ghadiri A.M. (2022). A Retrospective-Prospective Review of Suzuki–Miyaura Reaction: From Cross-Coupling Reaction to Pharmaceutical Industry Applications. Polyhedron.

[11] Dorel R., Grugel C.P., Haydl A.M. (2019). The Buchwald–Hartwig Amination after 25 Years. Angew. Chem. Int. Ed..

[12] Heravi M.M., Kheilkordi Z., Zadsirjan V., Heydari M., Malmir M. (2018). Buchwald-Hartwig Reaction: An Overview. J. Organomet. Chem..

[13] Zych D. (2019). Non-K Region Disubstituted Pyrenes (1,3-, 1,6- and 1,8-) by (Hetero)Aryl Groups—Review. Molecules.

[14] Dewar M.J.S., Dennington R.D.I. (1989). DEWAR-PI Study of Electrophilic Substitution in Selected Polycyclic Fluoranthene Hydrocarbons. J. Am. Chem. Soc..

[15] Cerfontain H., Laali K., Lambrechts H.J.A. (1983). Aromatic Sulfonation 86. Sulfonation of Pyrene, 1-Methylpyrene and Perylene. Recl. Trav. Chim. Pays-Bas.

[16] Vollmann H., Becker H., Corell M., Streeck H. (1937). Beiträge Zur Kenntnis Des Pyrens Und Seiner Derivate. Justus Liebigs Ann. Chem..

[17] Maeda H., Shoji T., Segi M. (2017). Effects of Substituents on Silicon Atoms upon Absorption and Fluorescence Properties of 1,3,6,8-Tetrakis(Silylethynyl)Pyrenes. Tetrahedron Lett..

[18] Bernhardt S., Kastler M., Enkelmann V., Baumgarten M., Müllen K. (2006). Pyrene as Chromophore and Electrophore: Encapsulation in a Rigid Polyphenylene Shell. Chem. A Eur. J.

[19] Zych D., Kurpanik A., Slodek A., Maroń A., Pająk M., Szafraniec-Gorol G., Matussek M., Krompiec S., Schab-Balcerzak E., Kotowicz S. (2017). NCN-Coordinating Ligands Based on Pyrene Structure with Potential Application in Organic Electronics. Chem. A Eur. J..

[20] Venkataramana G., Sankararaman S. (2005). Synthesis, Absorption, and Fluorescence-Emission Properties of 1,3,6,8-Tetraethynylpyrene and Its Derivatives. Eur. J. Org. Chem..

[21] Moorthy J.N., Natarajan P., Venkatakrishnan P., Huang D.-F., Chow T.J. (2007). Steric Inhibition of π-Stacking: 1,3,6,8-Tetraarylpyrenes as Efficient Blue Emitters in Organic Light Emitting Diodes (OLEDs). Org. Lett..

[22] Hu J., Hiyoshi H., Do J.-H., Yamato T. (2010). Synthesis and Fluorescence Emission Properties of 1,3,6,8-Tetrakis(9H-Fluoren-2-Yl)Pyrene Derivative. J. Chem. Res..

[23] Lock G. (1937). Über Abkömmlinge Des Pyrens. Berichte Dtsch. Chem. Ges. (A B Ser.).

[24] Schulze M. (2016). Synthesis of 1-Bromopyrene and 1-Pyrenecarbaldehyde. Org. Synth..

[25] He C., He Q., Chen Q., Shi L., Cao H., Cheng J., Deng C., Lin T. (2010). Highly Fluorescent Intramolecular Dimmers of Two Pyrenyl-Substituted Fluorenes Bridged by 1,6-Hexanyl: Synthesis, Spectroscopic, and Self-Organized Properties. Tetrahedron Lett..

[26] Wang T., Zhang N., Zhang K., Dai J., Bai W., Bai R. (2016). Pyrene Boronic Acid Cyclic Ester: A New Fast Self-Recovering Mechanoluminescent Material at Room Temperature. Chem. Commun..

[27] Chidirala S., Ulla H., Valaboju A., Kiran M.R., Mohanty M.E., Satyanarayan M.N., Umesh G., Bhanuprakash K., Rao V.J. (2016). Pyrene–Oxadiazoles for Organic Light-Emitting Diodes: Triplet to Singlet Energy Transfer and Role of Hole-Injection/Hole-Blocking Materials. J. Org. Chem..

[28] Yu L., Lo K.C., Xi J., Phillips D.L., Chan W.K. (2013). Photo-Induced Electron Transfer in a Pyrenylcarbazole Containing Polymer–Multiwalled Carbon Nanotube Composite. New J. Chem..

[29] Guo Y., Wang L., Zhuo J., Xu B., Li X., Zhang J., Zhang Z., Chi H., Dong Y., Lu G. (2017). A Pyrene-Based Dual Chemosensor for Colorimetric Detection of Cu^2+^ and Fluorescent Detection of Fe^3+^. Tetrahedron Lett..

[30] Harsha K.G., Appalanaidu E., Rao B.A., Baggi T.R., Rao V.J. (2020). ON–OFF Fluorescent Imidazole Derivative for Sensitive and Selective Detection of Copper(II) Ions. Russ. J. Org. Chem..

[31] Vyas P.V., Bhatt A.K., Ramachandraiah G., Bedekar A.V. (2003). Environmentally Benign Chlorination and Bromination of Aromatic Amines, Hydrocarbons and Naphthols. Tetrahedron Lett..

[32] Sharif M., Reimann S., Wittler K., Knöpke L.R., Surkus A., Roth C., Villinger A., Ludwig R., Langer P. (2011). 1-(Arylalkenyl)Pyrenes—Synthetic, Structural, Photophysical, Theoretical, and Electrochemical Investigations. Eur. J. Org. Chem..

[33] Reimann S., Sharif M., Wittler K., Knöpke L.R., Surkus A.-E., Roth C., Ludwig R., Langer P. (2013). 3-Pyrenylacrylates: Synthetic, Photophysical, Theoretical and Electrochemical Investigations. Z. Naturforschung B.

[34] Yang L., Finney N.S. (2017). A Mechanistically-Distinct Approach to Fluorescence Visualization of Singlet Oxygen. Chem. Commun..

[35] Kathayat R.S., Finney N.S. (2013). Sulfoxides as Response Elements for Fluorescent Chemosensors. J. Am. Chem. Soc..

[36] Đorđević L., Milano D., Demitri N., Bonifazi D. (2020). O-Annulation to Polycyclic Aromatic Hydrocarbons: A Tale of Optoelectronic Properties from Five- to Seven-Membered Rings. Org. Lett..

[37] Hsu C., Lin H., Yan X., Huang T., Su Y., Whang T. (2012). Fast Thermal Evaporation in Purification of 1,4-Di(Pyren-1-ly)Benzene. J. Chin. Chem. Soc..

[38] Doan B., Nguyen C., Bui T., Tran T., Huynh H., Nguyen Q.-T., Cu S., Nguyen L.-T., Tran C., Mai P. (2022). Synthesis of Conjugated Molecules Based on Dithienopyrrole Derivatives and Pyrene as Chemosensor for Mesotrione Detection. J. Braz. Chem. Soc..

[39] Keshtov M.L., Sharma G.D., Godovskii D.Y., Belomoina N.M., Geng Y., Zou Y., Kochurov V.S., Stakhanov A.I., Khokhlov A.R. (2014). Novel Electron-Withdrawing π-Conjugated Pyrene-Containing Poly(Phenylquinoxaline)s. Dokl Chem.

[40] Hu J.-Y., Feng X., Seto N., Do J.-H., Zeng X., Tao Z., Yamato T. (2013). Synthesis, Structural and Spectral Properties of Diarylamino-Functionalized Pyrene Derivatives via Buchwald–Hartwig Amination Reaction. J. Mol. Struct..

[41] Wu W., Wu X., Zhao J., Wu M. (2015). Synergetic Effect of C*N^N/C^N^N Coordination and the Arylacetylide Ligands on the Photophysical Properties of Cyclometalated Platinum Complexes. J. Mater. Chem. C.

[42] Mitchell R.H., Lai Y.-H., Williams R.V. (1979). N-Bromosuccinimide-Dimethylformamide: A Mild, Selective Nuclear Monobromination Reagent for Reactive Aromatic Compounds. J. Org. Chem..

[43] Kovalev I.S., Kopchuk D.S., Khasanov A.F., Zyryanov G.V., Rusinov V.L., Chupakhin O.N. (2014). The Synthesis of Polyarene-Modified 5-Phenyl-2,2′-Bipyridines via the Methodology and Aza-Diels–Alder Reaction. Mendeleev Commun..

[44] Zhang Q., Chan Y., Zhang M., Yeung Y., Ke Z. (2022). Hypervalent Chalcogenonium⋯π Bonding Catalysis. Angew. Chem. Int. Ed..

[45] He X., Wang X., Ke Z., Yeung Y.-Y. (2021). Bis-Selenonium Cations as Bidentate Chalcogen Bond Donors in Catalysis. ACS Catal..

[46] He X., Wang X., Tse Y., Ke Z., Yeung Y. (2018). Applications of Selenonium Cations as Lewis Acids in Organocatalytic Reactions. Angew. Chem. Int. Ed..

[47] Batabyal M., Upadhyay A., Kadu R., Birudukota N.C., Chopra D., Kumar S. (2022). Tetravalent Spiroselenurane Catalysts: Intramolecular Se···N Chalcogen Bond-Driven Catalytic Disproportionation of H_2_O_2_ to H_2_O and O_2_ and Activation of I_2_ and NBS. Inorg. Chem..

[48] Liu X., Luo G., Cai X., Wu H., Su S.-J., Cao Y. (2015). Pyrene Terminal Functionalized Perylene Diimide as Non-Fullerene Acceptors for Bulk Heterojunction Solar Cells. RSC Adv..

[49] Chow A.L., So M., Lu W., Zhu N., Che C. (2011). Synthesis, Photophysical Properties, and Molecular Aggregation of Gold(I) Complexes Containing Carbon-Donor Ligands. Chem. Asian J..

[50] Maeda H., Tanaka K., Aratani M., Segi M. (2019). Ethynylpyrene Linked Benzocrown Ethers as Fluorescent Sensors for Metal Ions. Photochem. Photobiol..

[51] Murai M., Ogita T., Takai K. (2019). Regioselective Arene Homologation through Rhenium-Catalyzed Deoxygenative Aromatization of 7-Oxabicyclo[2.2.1]Hepta-2,5-Dienes. Chem. Commun..

[52] Wang D., Jin Z., Tang J., Liang P., Mi Y., Miao Z., Zhang Y., Yang H. (2012). Photophysical and Self-Assembly Properties of Asymmetrical Multi-Aralkyl and Arylaldehyde Substituted Pyrene Derivatives. Tetrahedron.

[53] Yamato T., Fujimoto M., Nagano Y., Miyazawa A., Tashiro M. (1997). Electrophilic Substitution of 7- *tert* -Butyl-1-Substituted Pyrenes. A New Route for the Preparation of 1,3-Disubstituted Pyrenes. Org. Prep. Proced. Int..

[54] Niamnont N., Kimpitak N., Wongravee K., Rashatasakhon P., Baldridge K.K., Siegel J.S., Sukwattanasinitt M. (2013). Tunable Star-Shaped Triphenylamine Fluorophores for Fluorescence Quenching Detection and Identification of Nitro-Aromatic Explosives. Chem. Commun..

[55] Grimshaw J., Trocha-Grimshaw J. (1972). Characterisation of 1,6- and 1,8-Dibromopyrenes. J. Chem. Soc. Perkin Trans. 1.

[56] Bheemireddy S.R., Ubaldo P.C., Finke A.D., Wang L., Plunkett K.N. (2016). Contorted Aromatics via a Palladium-Catalyzed Cyclopentannulation Strategy. J. Mater. Chem. C.

[57] Gong X., Xie X., Chen N., Zheng C., Zhu J., Chen R., Huang W., Gao D. (2015). Two Symmetrically Bis-Substituted Pyrene Derivatives: Synthesis, Photoluminescence, and Electroluminescence. Chin. J. Chem..

[58] Minglun L., Gong X., Zheng C., Gao D. (2017). Development of Pyrene Derivatives as Promising N-Type Semiconductors: Synthesis, Structural and Spectral Properties. Asian J. Org. Chem..

[59] Lee H., Kim B., Kim S., Kim J., Lee J., Shin H., Lee J.-H., Park J. (2014). Synthesis and Electroluminescence Properties of Highly Efficient Dual Core Chromophores with Side Groups for Blue Emission. J. Mater. Chem. C.

[60] Kim B., Park Y., Lee J., Yokoyama D., Lee J.-H., Kido J., Park J. (2012). Synthesis and Electroluminescence Properties of Highly Efficient Blue Fluorescence Emitters Using Dual Core Chromophores. J. Mater. Chem. C.

[61] Lungerich D., Papaianina O., Feofanov M., Liu J., Devarajulu M., Troyanov S.I., Maier S., Amsharov K. (2018). Dehydrative π-Extension to Nanographenes with Zig-Zag Edges. Nat. Commun..

[62] Jung M., Lee J., Jung H., Park J. (2016). Synthesis and Physical Properties of New Pyrene Derivative with Bulky Side Groups for Blue Emission. J. Nanosci. Nanotechnol..

[63] Jung M., Lee J., Jung H., Kang S., Wakamiya A., Park J. (2018). Highly Efficient Pyrene Blue Emitters for OLEDs Based on Substitution Position Effect. Dye. Pigment..

[64] Gong X., Zheng C., Feng X., Huan Y., Li J., Yi M., Fu Z., Huang W., Gao D. (2018). 1,8-Substituted Pyrene Derivatives for High-Performance Organic Field-Effect Transistors. Chem. Asian J..

[65] Ðorđević L., Valentini C., Demitri N., Mézière C., Allain M., Sallé M., Folli A., Murphy D., Mañas-Valero S., Coronado E. (2020). O-Doped Nanographenes: A Pyrano/Pyrylium Route Towards Semiconducting Cationic Mixed-Valence Complexes. Angew. Chem. Int. Ed..

[66] Kim J.-H., Lee S., Kang I.-N., Park M.-J., Hwang D.-H. (2012). Photovoltaic Devices Using Semiconducting Polymers Containing Head-to-Tail-Structured Bithiophene, Pyrene, and Benzothiadiazole Derivatives. J. Polym. Sci. Part A Polym. Chem..

[67] Kim J.-H., Kim H., Kang I.-N., Lee S., Moon S.-J., Shin W.S., Hwang D.-H. (2012). Incorporation of Pyrene Units to Improve Hole Mobility in Conjugated Polymers for Organic Solar Cells. Macromolecules.

[68] Connor D.M., Kriegel R.M., Collard D.M., Liotta C.L., Schiraldi D.A. (2000). Pyrene and Anthracene Dicarboxylic Acids as Fluorescent Brightening Comonomers for Polyester. J. Polym. Sci. Part A Polym. Chem..

[69] Suenaga H., Nakashima K., Mizuno T., Takeuchi M., Hamachi I., Shinkai S. (1998). Pyrenylboronic Acids as a Novel Entry for Photochemical DNA Cleavage: Diradical-Forming Pyrene-1,6-Diyldiboronic Acid Mimics the Cleavage Mechanism of Enediyne Antitumor Antibiotics. J. Chem. Soc. Perkin Trans. 1.

[70] Kaplunov M.G., Yakushchenko I.K., Krasnikova S.S., Echmaev S.B. (2016). Novel 1,8-Bis(Diarylamino)Pyrenes as OLED Materials. Mendeleev Commun..

[71] Li R., Xu H., Zhang Y., Chang L., Ma Y., Hou Y., Miao S., Wang C. (2021). Electrochromic Properties of Pyrene Conductive Polymers Modified by Chemical Polymerization. RSC Adv..

[72] Zhang R., Zhao Y., Zhang T., Xu L., Ni Z.-H. (2016). A Series of Short Axially Symmetrically 1,3,6,8-Tetrasubstituted Pyrene-Based Green and Blue Emitters with 4-Tert-Butylphenyl and Arylamine Attachments. Dye. Pigment..

[73] Zhang R., Zhang T., Xu L., Han F., Zhao Y., Ni Z.-H. (2016). A New Series of Short Axially Symmetrically and Asymmetrically 1,3,6,8-Tetrasubstituted Pyrenes with Two Types of Substituents: Syntheses, Structures, Photophysical Properties and Electroluminescence. J. Mol. Struct..

[74] Arai R., Uemura S., Irie M., Matsuda K. (2008). Reversible Photoinduced Change in Molecular Ordering of Diarylethene Derivatives at a Solution−HOPG Interface. J. Am. Chem. Soc..

[75] Nakamura H., Tomonaga Y., Miyata K., Uchida M., Terao Y. (2007). Reaction of Polycyclic Aromatic Hydrocarbons Adsorbed on Silica in Aqueous Chlorine. Environ. Sci. Technol..

[76] Minabe M., Takeshige S., Soeda Y., Kimura T., Tsubota M. (1994). Electrophilic Substitution of Monosubstituted Pyrenes. Bull. Chem. Soc. Jpn..

[77] Gerasimenko Y.E., Poteleshchenko V.P. (1972). Some substituted pyrene-2-carboxylic acid. J. Org. Chem. USSR.

[78] Nielsen T., Siigur K., Helweg C., Jørgensen O., Hansen P.E., Kirso U. (1997). Sorption of Polycyclic Aromatic Compounds to Humic Acid As Studied by High-Performance Liquid Chromatography. Environ. Sci. Technol..

[79] Casas-Solvas J., Mooibroek T., Sandramurthy S., Howgego J., Davis A. (2014). A Practical, Large-Scale Synthesis of Pyrene-2-Carboxylic Acid. Synlett.

[80] Gooßen L.J., Thiel W.R., Rodríguez N., Linder C., Melzer B. (2007). Copper-Catalyzed Protodecarboxylation of Aromatic Carboxylic Acids. Adv. Synth. Catal..

[81] Stock L.M., Obeng M. (1997). Oxidation and Decarboxylation. A Reaction Sequence for the Study of Aromatic Structural Elements in Pocahontas No. 3 Coal. Energy Fuels.

[82] Goossen L.J., Manjolinho F., Khan B.A., Rodríguez N. (2009). Microwave-Assisted Cu-Catalyzed Protodecarboxylation of Aromatic Carboxylic Acids. J. Org. Chem..

[83] Maeda H., Hironishi M., Ishibashi R., Mizuno K., Segi M. (2017). Synthesis and Fluorescence Properties of Dioxa-, Dithia-, and Diselena-[3.3](1,3)Pyrenophanes. Photochem. Photobiol. Sci..

[84] Li Y.F., Xie X., Gong X.J., Liu M.L., Chen R.F., Gao D.Q., Huang W. (2017). Two Bipolar Blue-Emitting Fluorescent Materials Based on 1,3,5-Triazine and Peripheral Pyrene for Organic Light-Emitting Diodes. Dye. Pigment..

[85] Ding Z., Ma B., Zhou Z., Zhang S., Pan J., Zhu W., Liu Y. (2023). Efficient Solution-Processed OLEDs Featuring Deep-Blue Triplet-Triplet Annihilation Emitter Based on Pyrene Derivative. Dye. Pigment..

[86] Feng X., Hu J.-Y., Tomiyasu H., Tao Z., Redshaw C., Elsegood M.R.J., Horsburgh L., Teat S.J., Wei X.-F., Yamato T. (2015). Iron(III) Bromide Catalyzed Bromination of 2-Tert-Butylpyrene and Corresponding Position-Dependent Aryl-Functionalized Pyrene Derivatives. RSC Adv..

[87] Feng X., Hu J.-Y., Yi L., Seto N., Tao Z., Redshaw C., Elsegood M.R.J., Yamato T. (2012). Pyrene-Based Y-Shaped Solid-State Blue Emitters: Synthesis, Characterization, and Photoluminescence. Chem. Asian J..

[88] Feng X., Iwanaga F., Hu J.-Y., Tomiyasu H., Nakano M., Redshaw C., Elsegood M.R.J., Yamato T. (2013). An Efficient Approach to the Synthesis of Novel Pyrene-Fused Azaacenes. Org. Lett..

[89] Figueira-Duarte T.M.M., Simon S.C.C., Wagner M., Druzhinin S.I.I., Zachariasse K.A.A., Müllen K. (2008). Polypyrene Dendrimers. Angew. Chem. Int. Ed..

[90] Hashikawa Y., Murata M., Wakamiya A., Murata Y. (2017). Palladium-Catalyzed Cyclization: Regioselectivity and Structure of Arene-Fused C60 Derivatives. J. Am. Chem. Soc..

[91] Wang C.-Z., Ichiyanagi H., Sakaguchi K., Feng X., Elsegood M.R.J., Redshaw C., Yamato T. (2017). Pyrene-Based Approach to Tune Emission Color from Blue to Yellow. J. Org. Chem..

[92] Lorbach D., Keerthi A., Figueira-Duarte T.M., Baumgarten M., Wagner M., Müllen K. (2016). Cyclization of Pyrene Oligomers: Cyclohexa-1,3-Pyrenylene. Angew. Chem. Int. Ed..

[93] Sun H., Wei J.-H., Xu L.-H., Jiang Y., Miao B.-X., Ni Z.-H. (2020). Temperature- and Solvent-Induced Solid-State Emission Changes and AIEE Property of a Pyrene-Based Sulfide Compound. Dye. Pigment..

[94] Wang C.-Z., Pang Z.-J., Yu Z.-D., Zeng Z.-X., Zhao W.-X., Zhou Z.-Y., Redshaw C., Yamato T. (2021). Short Axially Asymmetrically 1,3-Disubstituted Pyrene-Based Color-Tunable Emitters: Synthesis, Characterization and Optical Properties. Tetrahedron.

[95] Li F., Pan M., He Q., Zhou Q., Tang Q., Gong C. (2022). Ester-Functionalized Pyrene Derivatives: Effects of Ester Substituents on Photophysical, Electrochemical, Electrochromic, and Electrofluorochromic Properties. Dye. Pigment..

[96] Idzik K.R., Licha T., Lukeš V., Rapta P., Frydel J., Schaffer M., Taeuscher E., Beckert R., Dunsch L. (2014). Synthesis and Optical Properties of Various Thienyl Derivatives of Pyrene. J. Fluoresc..

[97] Idzik K.R., Cywiński P.J., Kuznik W., Frydel J., Licha T., Ratajczyk T. (2015). The Optical Properties and Quantum Chemical Calculations of Thienyl and Furyl Derivatives of Pyrene. Phys. Chem. Chem. Phys..

[98] Maeda H., Ueno R., Furuyama T., Segi M. (2020). Effects of Substituents on Absorption and Fluorescence Properties of Trimethylsilylethynyl- and Tert-Butylethynyl-Pyrenes. J. Photochem. Photobiol. A Chem..

[99] Thakur K., Wang D., Mirzaei S., Rathore R. (2020). Electron-Transfer-Induced Self-Assembly of a Molecular Tweezer Platform. Chem. A Eur. J..

[100] Ahn Y., Kim S., Song J.H., Yeom W., Lee J., Suh M.C. (2022). The Positional Effect of Arylamines on Pyrene Core in a Blue Fluorescent Dopant Significantly Affecting the Performance of Organic Light Emitting Diodes. Dye. Pigment..

[101] Streitwieser A., Lawler R.G., Schwaab D. (1965). On the Bromopyrenes1. J. Org. Chem..

[102] Lee H., Harvey R.G. (1986). Synthesis of 2,7-Dibromopyrene. J. Org. Chem..

[103] Foroozesh M., Primrose G., Guo Z., Bell L.C., Alworth W.L., Guengerich F.P. (1997). Aryl Acetylenes as Mechanism-Based Inhibitors of Cytochrome P450-Dependent Monooxygenase Enzymes. Chem. Res. Toxicol..

[104] Harvey R.G., Schmolka S., Cortez C., Lee H. (1988). Syntheses of 2-Bromopyrene and 2-Hydroxypyrene. Synth. Commun..

[105] El-Ballouli A.O., Khnayzer R.S., Khalife J.C., Fonari A., Hallal K.M., Timofeeva T.V., Patra D., Castellano F.N., Wex B., Kaafarani B.R. (2013). Diarylpyrenes vs. Diaryltetrahydropyrenes: Crystal Structures, Fluorescence, and Upconversion Photochemistry. J. Photochem. Photobiol. A Chem..

[106] Godinez C.E., Zepeda G., Garcia-Garibay M.A. (2002). Molecular Compasses and Gyroscopes. II. Synthesis and Characterization of Molecular Rotors with Axially Substituted Bis[2-(9-Triptycyl)Ethynyl]Arenes. J. Am. Chem. Soc..

[107] Zhang Y., Tan L., Shi J., Ji L. (2021). Iridium-Catalysed Borylation of Pyrene—A Powerful Route to Novel Optoelectronic Materials. New J. Chem..

[108] Cirera B., Đorđević L., Otero R., Gallego J.M., Bonifazi D., Miranda R., Ecija D. (2016). Dysprosium-Carboxylate Nanomeshes with Tunable Cavity Size and Assembly Motif through Ionic Interactions. Chem. Commun..

[109] Crawford A.G., Liu Z., Mkhalid I.A.I., Thibault M.-H., Schwarz N., Alcaraz G., Steffen A., Collings J.C., Batsanov A.S., Howard J.A.K. (2012). Synthesis of 2- and 2,7-Functionalized Pyrene Derivatives: An Application of Selective C-H Borylation. Chemistry.

[110] Mitchell R.H., Chen Y., Zhang J. (1998). ChemInform Abstract: N-Bromosuccinimide—Chloroform, a More Convenient Method to Nuclear Brominate Reactive Aromatic Hydrocarbons. ChemInform.

[111] Sasaki S., Suzuki S., Igawa K., Morokuma K., Konishi G. (2017). The K-Region in Pyrenes as a Key Position to Activate Aggregation-Induced Emission: Effects of Introducing Highly Twisted *N*,*N*-Dimethylamines. J. Org. Chem..

